# Incidence of late-onset hemorrhagic cystitis and its effect on PFS in acute leukemia patients after haplo-PBSCT: The 5-year single-center data

**DOI:** 10.3389/fonc.2022.913802

**Published:** 2022-07-15

**Authors:** Hailong Yuan, Gang Chen, Jianli Xu, Ruixue Yang, Maria Muhashi, Gulibadanmu Aizezi, Ming Jiang

**Affiliations:** Hematology Center, The First Affiliated Hospital of Xinjiang Medical University, Xinjiang Institute of Hematology, Urumqi, China

**Keywords:** hemorrhagic cystitis, late-onset hemorrhagic cystitis, allo-genetic hematopoietic stem cell transplantation, haploid, progression free survival (PFS)

## Abstract

We conducted a single-center 5-year retrospective study on the occurrence of hemorrhagic cystitis (HC) and its effect on survival after haploid high-dose peripheral blood stem cell transplantation (haplo-PBSCT) in patients with acute leukemia. We retrospectively analyzed 153 patients with acute leukemia who were treated with non-*in vitro* T-cell depleted haplo-PBSCT and myeloablative conditioning regimen. All patients were followed up for more than 180 days after transplantation. HC occurrence and its effect on long-term progression free survival (PFS) were retrospectively analyzed. Totally, 64 out of 153 patients had late onset HC (LOHC). No early onset HC occurred. The median onset time was 38.5 (17-163) days after transplantation. The cumulative incidence of LOHC was 41.8%. The cumulative incidence of LOHC in patients under 27 years old (50.0%) and in ALL patients (54.1%) was significantly higher than that in patients over 27 years old (34.5%) and in AML patients (36.9%), respectively. The cumulative incidence of mild LOHC was 44.2% and that of severe LOHC was 28.6%. However, urine copies of BK virus were not related to LOHC duration. There was no significant difference in 3-year expected PFS between AML and ALL patients with and without LOHC, or between LOHC duration more than and less than 38.5 days (P>0.05). Conclusively, LOHC incidence is higher in patients under 27 years old and in ALL patients. LOHC occurrence is related to urine BK virus copy, but not blood BK virus load. LOHC duration and severity has no significant effect on PFS.

## Introduction

Hemorrhagic cystitis (HC) is one of the more common and serious complications after allogenetic hematopoietic stem cell transplantation (allo-HSCT). The main manifestation is hematuria, including microscopic hematuria, severe bleeding, blood clots in the urine, and even urinary tract obstruction (1). HC seriously affects the quality of life of patients, significantly increases the length of hospitalization and medical expenses of patients, and even causes renal function damage that requires surgical intervention (2). Therefore, HC is still one of the main complications that need attention after HSCT.

The occurrence of early onset HC (EOHC) is mainly related to the use of large doses of cyclophosphamide (Cy), which usually occurs within 24 to 72 h after conditioning. Acrolein, a metabolite of Cy, is highly toxic to the urothelium, the effect of which can be alleviated by mesna, thereby reducing the occurrence of HC (3, 4). The occurrence of EOHC has been significantly reduced after application of mesna and comprehensive preventive measures such as fluid replacement and diuresis. However, LOHC occurred 72 h after conditioning. It is currently believed that BK virus infection is the main cause of LOHC, and HLA incompatible transplantation, graft-versus-host disease (GVHD) and cytomegaovirus (CMV) are also important risk factors for the occurrence of LOHC (5–7). The incidence of LOHC after HSCT is 7% to 68%, and the incidence of severe LOHC is 29%-44% (8–10). The incidence of LOHC is higher after haploidentical peripheral blood stem cell transplantation (haplo-PBSCT), which is about 2-3 times that of identical transplantation (11). It is shown that LOHC may increase transplant-related mortality (TRM), but its effect on overall survival (OS) is limited (12). However, other studies have shown that LOHC related with BK virus infection reduces OS of patients (2, 13). Therefore, whether LOHC has a significant effect on patient survival after transplantation is still controversial.

Previously, we showed that the incidence of LOHC increased after haplo-PBSCT, but LOHC had no significant effect on the progression-free survival (PFS) of patients after transplantation (14). Here, in this study, we further investigated the incidence of LOHC and the effect of LOHC on PFS of patients with acute leukemia after haplo-PBSCT using 5-year single-center data. We retrospectively analyzed 153 cases with acute leukemia over 14 years old who received myeloablative haplo-PBSCT in our transplant center from January 1, 2015, to December 31, 2020. All patients have been followed up for more than 180 days. Under the anti-thymocyte globulin (ATG)-based myeloablative transplantation model, we further analyzed the occurrence characteristics of LOHC, the relationship between LOHC and BK virus, and the effect of LOHC severity and duration and disease type on PFS of patients. Our results may provide a reference for further studies on the occurrence characteristics of LOHC and its effect on PFS after Haplo-PBSCT transplantation.

## Methods

### Patients

We included patients with acute leukemia who underwent haplo-PBSCT at the transplant center from January 1, 2015, to December 31, 2020. The inclusion criteria were: 1) Patients aged from 14 to 55 years old; 2) Patients with no obvious damage in other important organs, such as heart and lungs; 3) Patients with HLA matched by high-resolution genotyping, including 10 alleles (HLA-A, B, DRB1), with 4/10 to 6/10 mismatch; 4) Patients with acute leukemia; 5) Patients were followed up for more than 180 days. The exclusion criteria were: 1) Patients < 14-year old, or > 55-year-old; 2) Patients with active infection; 3) Patients with damage in heart, lung, kidney or liver. This study was approved by the ethics committee of Xinjiang Medical University and all methods were also performed in accordance with the ethical standards as laid down in the 1964 Declaration of Helsinki and its later amendments or comparable ethical standards. Written informed consent were obtained from all patients or their legal guardians.

### Follow-up

All patients were followed up through outpatient, hospitalization, telephone, etc. The deadline for follow-up was July 1, 2021. All patients were followed up for more than 180 days, with the median follow-up time of 20 (96-77.5) months. No patients were lost to follow-up.

### Myeloablative conditioning (MAC) conditioning regimen

The MAC regimen was Cytarabine+ Busulfan/Cy regimen based on ATG. In detail, cytarabine was administered at 2-4 g·m^-2^·d^-1^ (day -9 to day -8). Busulfan was administered at 3.2 mg·kg^-1^·d^-1^ (day -7 to day -5). Cy was administered at 1.8 g·m^-2^·d^-1^ (day -3 to day -2). ATG at 2.5 mg·kg^-1^·d^-1^ (day -4 to day -1) was administered.

### Prevention of GVHD

CsA or Tac + short-term MTX + MMF + Glu was used as the basis. CsA was performed -5d~+100d, with starting dose 2~2.5mg/kg·d intravenously, and then 4~5mg/kg·d orally; or, Tac -5d ~ +100d, with initial dosage 0.02 mg/kg·d intravenously, then 0.05 mg/kg·d orally twice; MMF 0.5 bid po, -1d~+100d; MTX 15 mg/m^2^·d, +1d; 10 mg/m^2^·d, +3d, +6d, +11d ivgtt; anti-CD25 monoclonal antibody (12 mg/m^2^) 20 mg ivgtt qd, 01 d and +2d; and +1~+15d after transplantation. Dexamethasone 2.5 mg was given intravenously, which was gradually change to oral prednisone. Oral prednisone was stopped within 2-3 weeks according to the patient’s condition.

### Stem cell collection from the donors

Stem cells were derived from peripheral blood induced by G-CSF (5-10 µg/kg/d, starting at -4d). On 1d and 2d, 2-3 TBV of peripheral blood stem cells were collected and infused into the patients *via* central vein (mononuclear cells (MNC) 12-20×10^8^/kg or CD34+ cells ≥8×10^6^/kg). If the donor’s body weight is less than 5 kg or more than the patient, and it is estimated that insufficient MNC or CD34+ cells can be obtained, stem cells from the donor will be collected for 3 days.

### Prevention of virus infection

Ganciclovir (250 mg ivgtt b.i.d) was used for pre-treatment from -7d to -1d. Acyclovir (250 mg ivgtt b.i.d) was used for further treatment on +1 day until the patients’ neutrophils increased to more than 0.5×10^9^/L. After that, ganciclovir (250 mg ivgtt b.i.d) was used again on +15d, and it was continuously used for about 2 weeks according to the patients’ blood routine.

### HC prevention

During the Cy period, the patients were given a large amount of fluid replacement for 24 h and subjected to alkalized urine and diuresis treatment. Mesna was used to prevent HC. Specifically, 1) Large-dose fluid replacement. The daily total fluid volume was calculated as 100~120 ml/kg/d, and the fluid replacement was performed intravenously and continuously. 2) Alkalized urine. The amount of sodium bicarbonate was 0.5% of the total fluid replacement. 3) Diuresis. The Furosemide injection was given at 20 mg/time and once every 6 h. The dosage and frequency can be adjusted according to the patient’s symptoms and electrolytes. Potassium supplementation was performed at the same time, and the amount of potassium chloride was 1.5% of the total fluid replacement. 4) The dosage of Mesna (sodium thioethanesulfonate) was 1.2 times that of CTX. The first dose of Mesna was 20% of CTX, and it was used simultaneously with CTX. The remaining mesna was given intravenously in 24 h.

### HC examination and grading

HC was examined by urinary tract ultrasound (for males), gynecological ultrasound (for females), urine routine test, urine bacterial and fungal culture, and cystoscopy if necessary. HC was graded as I (microscopic hematuria), II (gross hematuria), III (gross hematuria with blood clots), and IV (gross hematuria, blood clots, and urinary tract obstruction). Degrees I~II were mild HC, and degrees III~IV were severe HC (15).

### Virus detection after HC occurrence

After HC occurrence, the CMV antibody, blood BK virus and JC virus in the blood were tested. The nucleic acid of CMV, BK virus and JC virus in the urine was detected by PCR. Meanwhile, BK and JC viruses in the blood and urine were tested in patients without HC during the same period.

### General treatment of HC

1) Fluid replacement, diuresis, and urine alkalization treatments were given to patients with HC. 2) The ribavirin or acyclovir was empirically used for antiviral therapy. 3) For patients with infection of BK virus and other viruses, the immunosuppressants (especially glucocorticoids) were reduced or stopped for 1 week or 2 weeks, and acyclovir or cyclovir antiviral therapy was actively performed. If there is a decrease in blood cells, ganciclovir can also be replaced with foscarnet. 4) Treatment with human immunoglobulin (0.4 g/kg, 3-4 days) was performed. 5) For patients without virus infection, on the basis of replacement, human immunoglobulin and other treatments, intravenous infusion of methylprednisolone or dexamethasone may be considered for patients with severe HC. 6) Adipose-derived mesenchymal stem cells (ADSCs) were used for treatment of severe HC. If there is no improvement after antiviral, fluid replacement, and diuretic treatment in patients with severe HC, ADSCs treatment was performed. Each infusion of ADSCs was at the dose of 1×10^6^/kg and once a week. The curative effect of ADSCs was evaluated after 3 infusions.

### Statistical analysis

SPSS26.0 statistical software was used for statistical analysis. The t test was used to analyze the statistical differences, such as those in the HC onset time, duration, patient age and virus copy number. Kaplan-Merier method was used for LOHC incidence and survival analysis. Log-rank test was performed to analyze the difference in survival. P <0.05 is considered as significant difference. All reported P values were based on two-sided tests.

## Results

### Baseline characteristics of the patients

Totally, we enrolled 153 patients with acute leukemia. Patient characteristics are summarized in **Table 1**. There were 98 males and 55 females, with a median age of 28 years (14-55 years). According to disease type, there were 89 cases of acute myeloid leukemia (AML) (80 cases of high risk and 9 cases of intermediate risk), and 64 cases of acute lymphocyte (ALL) (58 cases of high risk and 6 cases of standard risk). All patients received MAC. Five patients with severe LOHC lasting more than 1 month were treated with ADSCs.

**Table 1:** Basic characteristics of patients.

Variable	NO. (%)
Sex	
Male	98(64)
Female	55(36)
Median age (year, range)	28(14-55)
14 – 28	71(46.4)
>28	82(53.6)
Disease	
AML	89(58.2)
High risk	80(89.9)
Intermediate risk	9(10.1)
ALL	64(41.8)
High risk	58(90.6)
Standard risk	6(9.4)
Donor-patient sex match	
Male to Male	42(27.4)
Male to Female	32(21)
Female to Male	56(36.6)
Female to Femal	23(15)
Donor-patient relationship	
Mother to child	37(24.2)
Father to child	34(22.2)
Child to patient	34(22.2)
Sibling	48(31.4)
ABO match	
Matched	79(51.6)
Minor mismatched	41(26.8)
Major mismatched	33(21.6)
ADSCs infusion	5(7.8)

AML, acute myeloid leukemia; ALL, acute lymphoblastic leukemia; ADSCs: Adipose-derived mesenchymal stem cells.

### Evidence of hematopoietic reconstruction and engraftment

All 153 patients had engraftment after haplo-PBSCT. The median number of mononuclear cells transplanted in patients was 12.1 (8.05~18.9) × 10^8^/kg, and the median number of CD34+ cells was 7.9 (5.3~15.4) × 10^6^/kg. The median time for neutrophils to be ≥ 0.5×10^9^/L was 14 (12~32) days. The median time for blood platelet count ≥ to be 20×10^9^/L in 7 days without platelet transfusion was 17 (14~34) days.

### LOHC and virus infection

Among the 153 patients, 64 patients developed LOHC after transplantation, but no patient had EOHC. The median LOHC onset time was 20.5 (9-141) days. Among the 64 patients with LOHC, 52 patients were tested for BK and JC viruses. The results showed that the JC virus detection in blood was negative, and only two patients had BK virus copy numbers in the blood higher than the reference value. In the urine samples, 36 cases had JC virus copy number lower than the reference value (less than 2000 copies/ml), and 16 cases had increased JC virus copy number (mean 2.38×10^7^ copies/ml and range 1.5×10^5^-9.6×10^8^ copies/ml). The copy number of BK virus in the urine in 5 cases was lower than the reference value (less than 2000 copies/ml), and that of 47 cases was increased (mean 1.92×10^8^ copies/ml and range 1.28×10^4^-8.96×10^8^ copies/ml). These results indicate that the occurrence of LOHC may be related to BK virus in the urine. In addition, during the diagnosis of HC, the bivariate correlation analysis showed that there was no correlation between BK virus copy number in the urine and LOHC duration (Spearman correlation coefficient: r=0.067, P=0.638).

### Analysis of LOHC incidence

**Figure 1:**
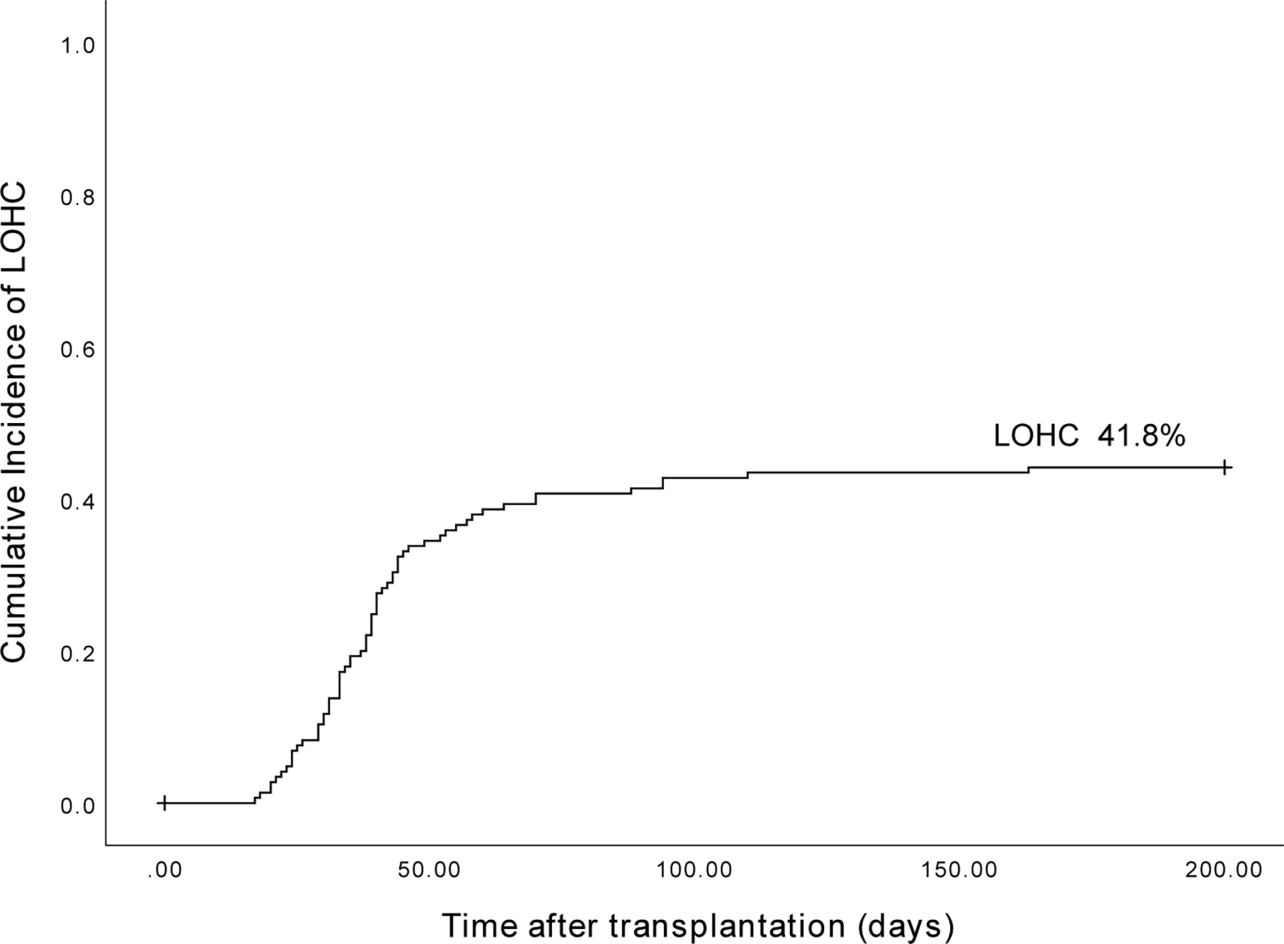
The cumulative incidence of LOHC in 153 patients after haplo-PBSCT was 41.8% (95%CI, 33.96%~66.04%).

**Figure 2:**
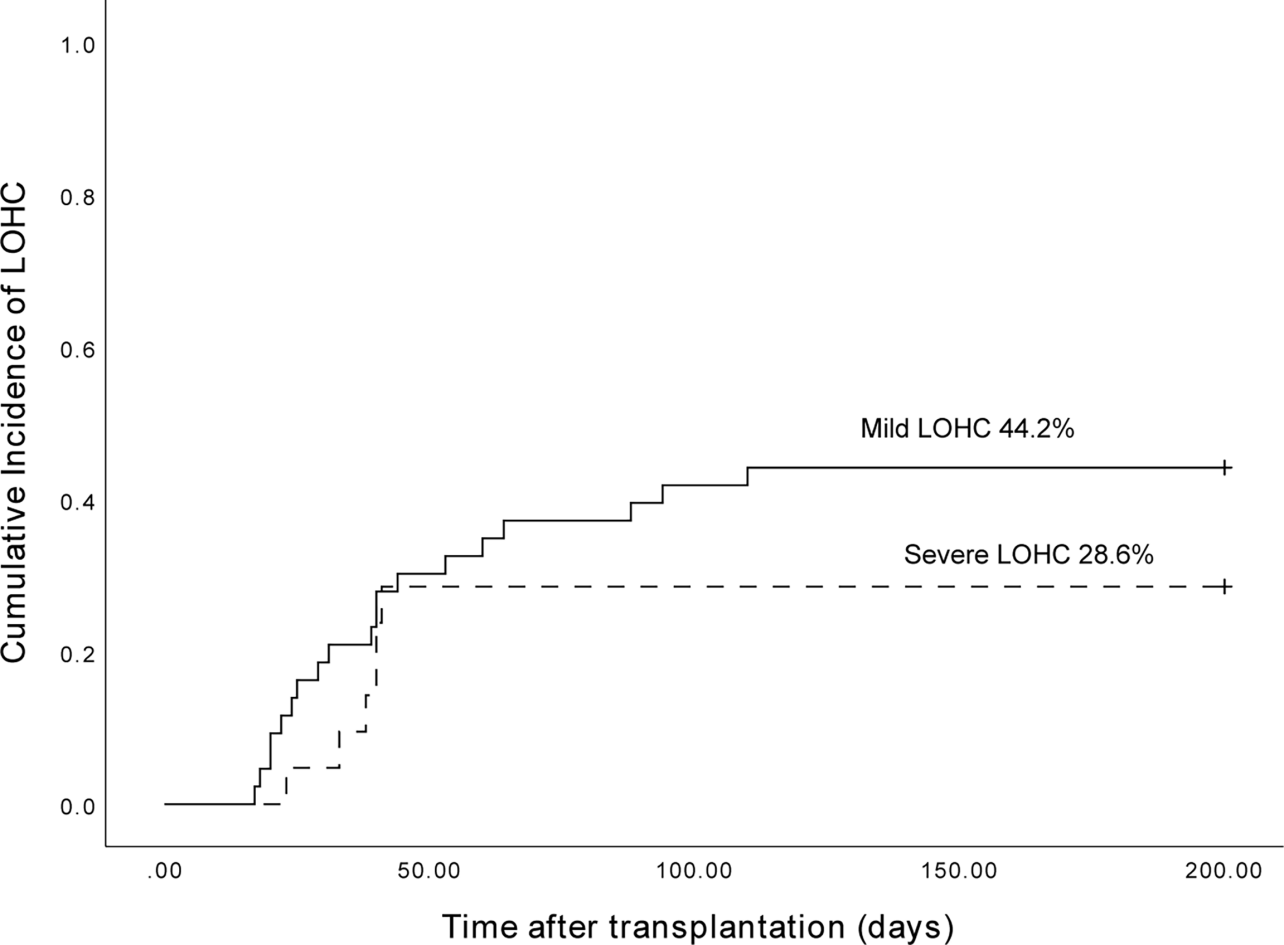
The cumulative incidence of mild LOHC and severe LOHC was 44.2% (95%CI, 29.4%~59%) and 28.6% (95%CI, 9.2%~48%), respectively.

The cumulative incidence of LOHC in 153 patients after transplantation was 41.8% (95%CI, 33.96%~66.04%) (**Figure 1**). In total, there were 43 cases of mild LOHC (9 cases of degree I LOHC and 34 cases of degree II LOHC) and 21 cases of severe LOHC (18 cases of degree III LOHC and 3 cases of degree IV LOHC). The cumulative incidence of mild LOHC was 44.2% (95%CI, 29.4%~59%), significantly lower than that of severe LOHC (41.8% (95%CI, 33.96%~66.04%); P=0.048) (**Figure 2**).

**Figure 3:**
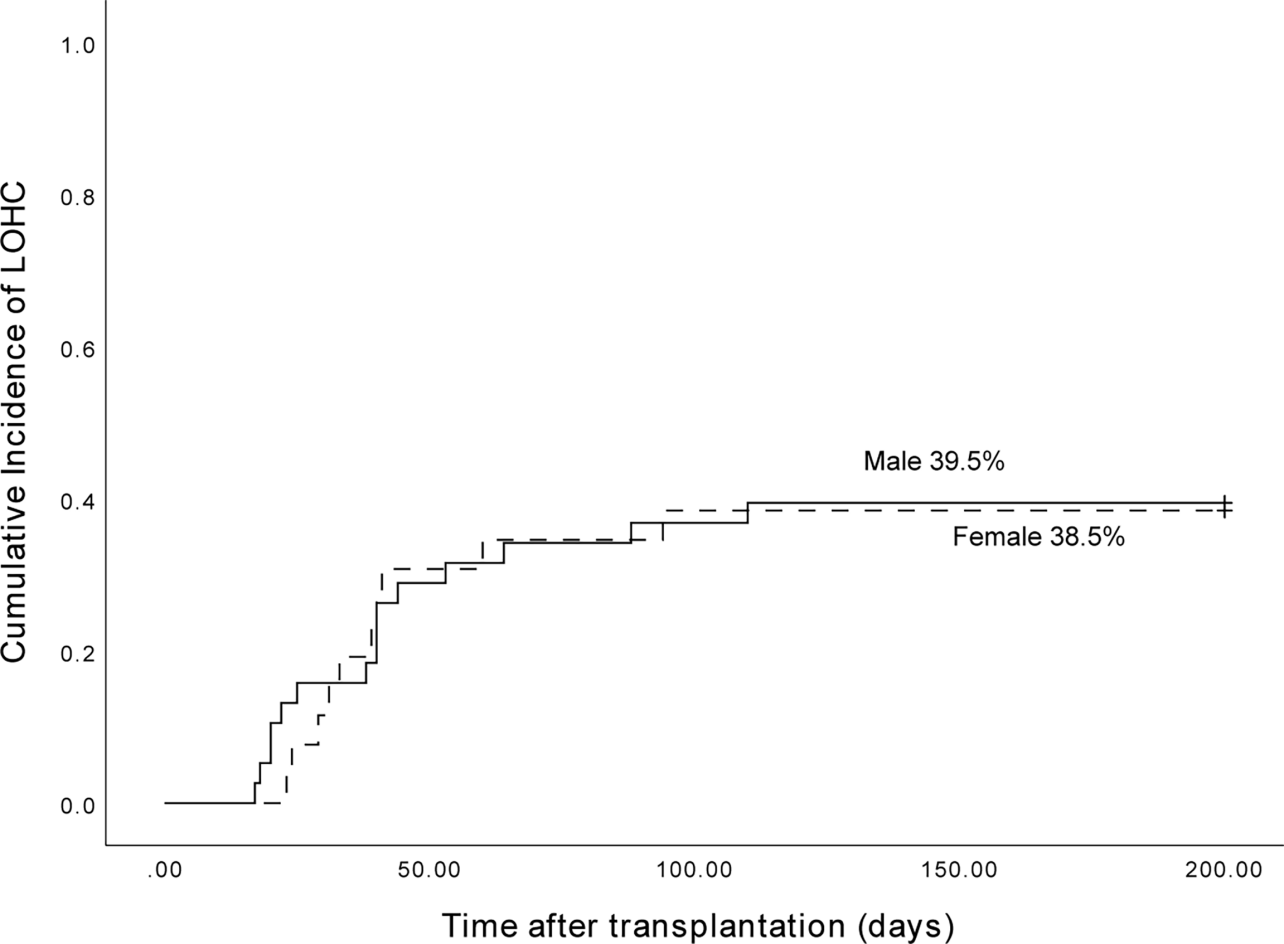
The cumulative incidence of LOHC in male and female patients was 39.5% (95%CI, 24.02%~54.98%) and 38.5% (95%CI, 19.49%~57.5%), respectively.

Among 153 patients, 38 of 98 male patients developed LOHC, and 26 of 55 female patients developed LOHC. The cumulative incidence of LOHC in males was 39.5% (95%CI, 24.02%~54.98%) and in females was 38.5% (95%CI, 19.49%~57.5%) (**Figure 3**). The incidence of LOHC in male patients was slightly higher than that in females, but the difference was not statistically significant (p=0.698).

**Figure 4:**
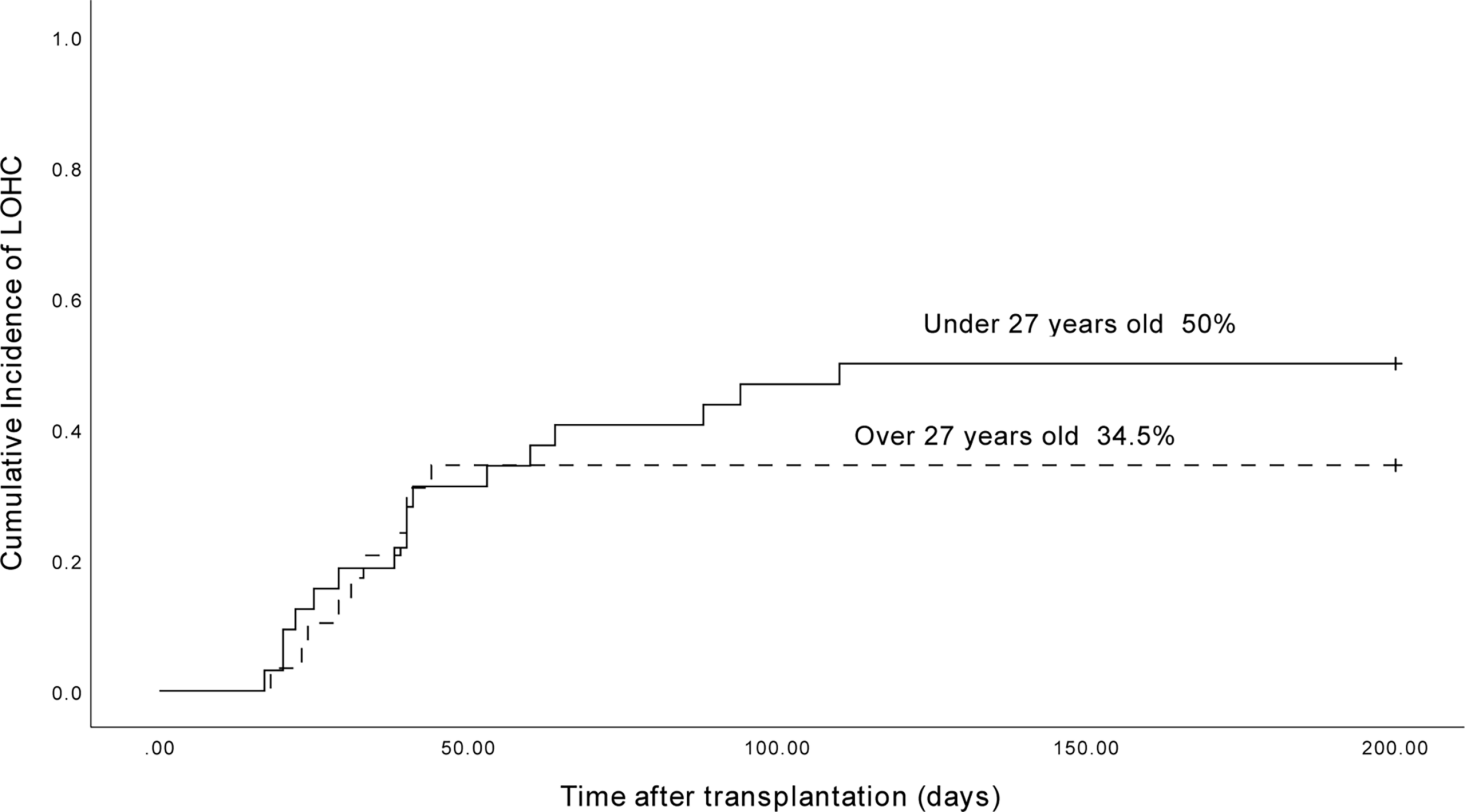
The cumulative incidence of LOHC in patients under 27-year-old and above 27-year-old was 50.0% (95%CI, 32.8%~67.2%) and 34.5% (95%CI, 17.3%~51.7%), respectively.

The median age of 64 LOHC patients was 26.8 years old (14 to 54 years old). The cumulative incidence of LOHC in patients under 27 years old was 50.0% (95% CI, 32.8% to 67.2%), which was significantly higher than that in patients over 27 years old (34.5% (95%CI, 17.3%~51.7%); p=0.048) (**Figure 4**).

**Figure 5:**
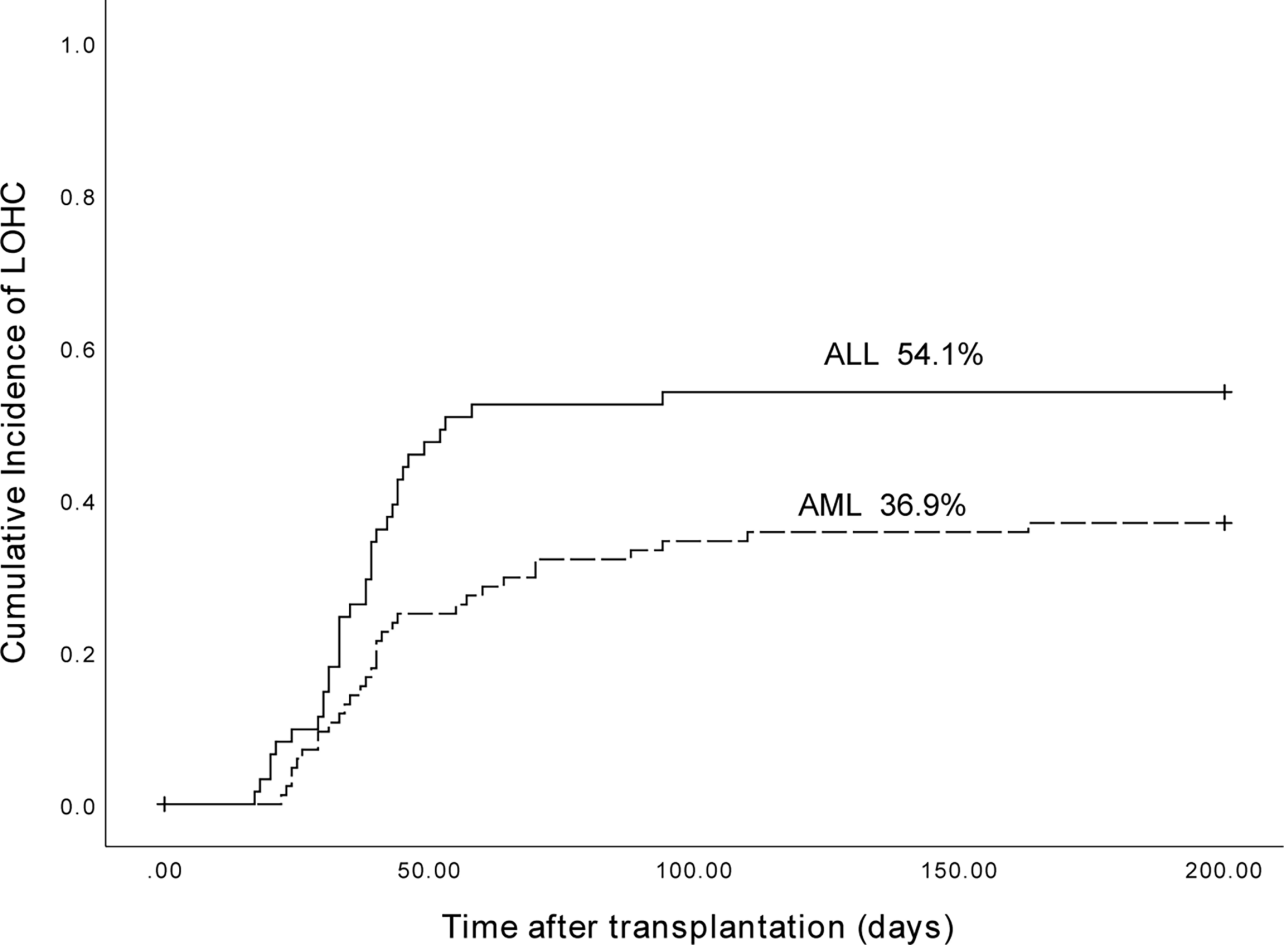
The cumulative incidence of LOHC in AML and ALL patients was 36.9% (95%CI, 26.51%~47.29%) and 54.1% (95%CI, 41.56%~66.64%), respectively (p=0.022).

Of the 153 patients, 89 were AML patients, and 31 had LOHC. There were 64 patients with ALL, and 33 patients developed LOHC. The cumulative incidence of LOHC in AML patients was 36.9% (95%CI, 26.51%~47.29%), significantly lower than that in ALL patients (54.1% (95%CI, 41.56%~66.64%); p=0.022) (**Figure 5**).

### Relationship between LOHC and PFS of AML and ALL patients

**Figure 6:**
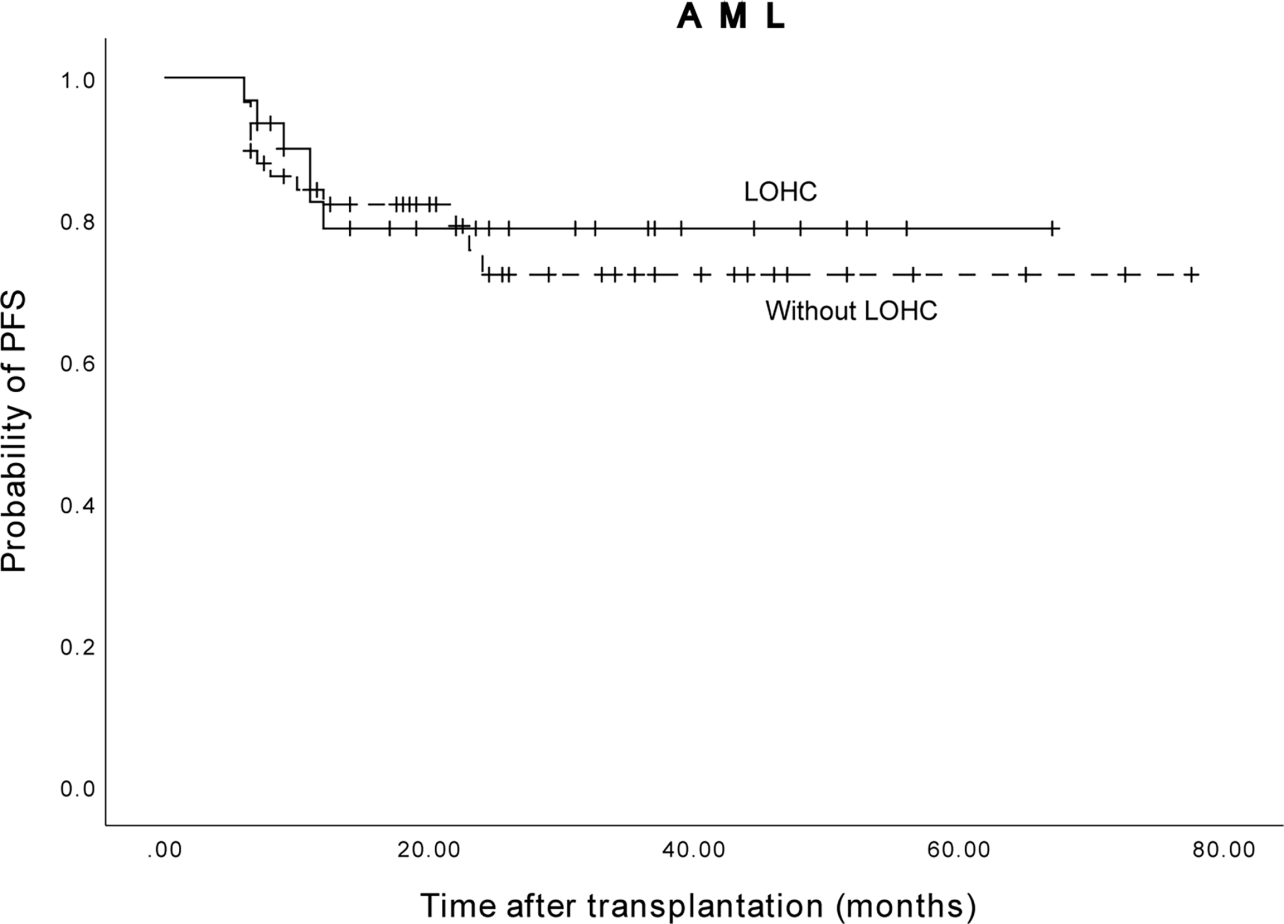
The three years expected PFS of AML patients with and without LOHC was 78.7% (95%CI, 63.41%~93.99%) and 72.2% (95%CI, 58.48%~85.92%), respectively.

Among 89 AML patients, the 3-year expected PFS of 58 patients without LOHC was 72.2% (95%CI, 58.48%~85.92%), and the 3-year expected PFS of 31 patients with LOHC was 78.7% (95%CI, 63.41%~93.99%) (**Figure 6**). There was no significant difference in PFS between AML patients with and without LOHC (p=0.951). It is suggested that LOHC in AML patients does not significantly affect the PFS of patients after transplantation.

**Figure 7:**
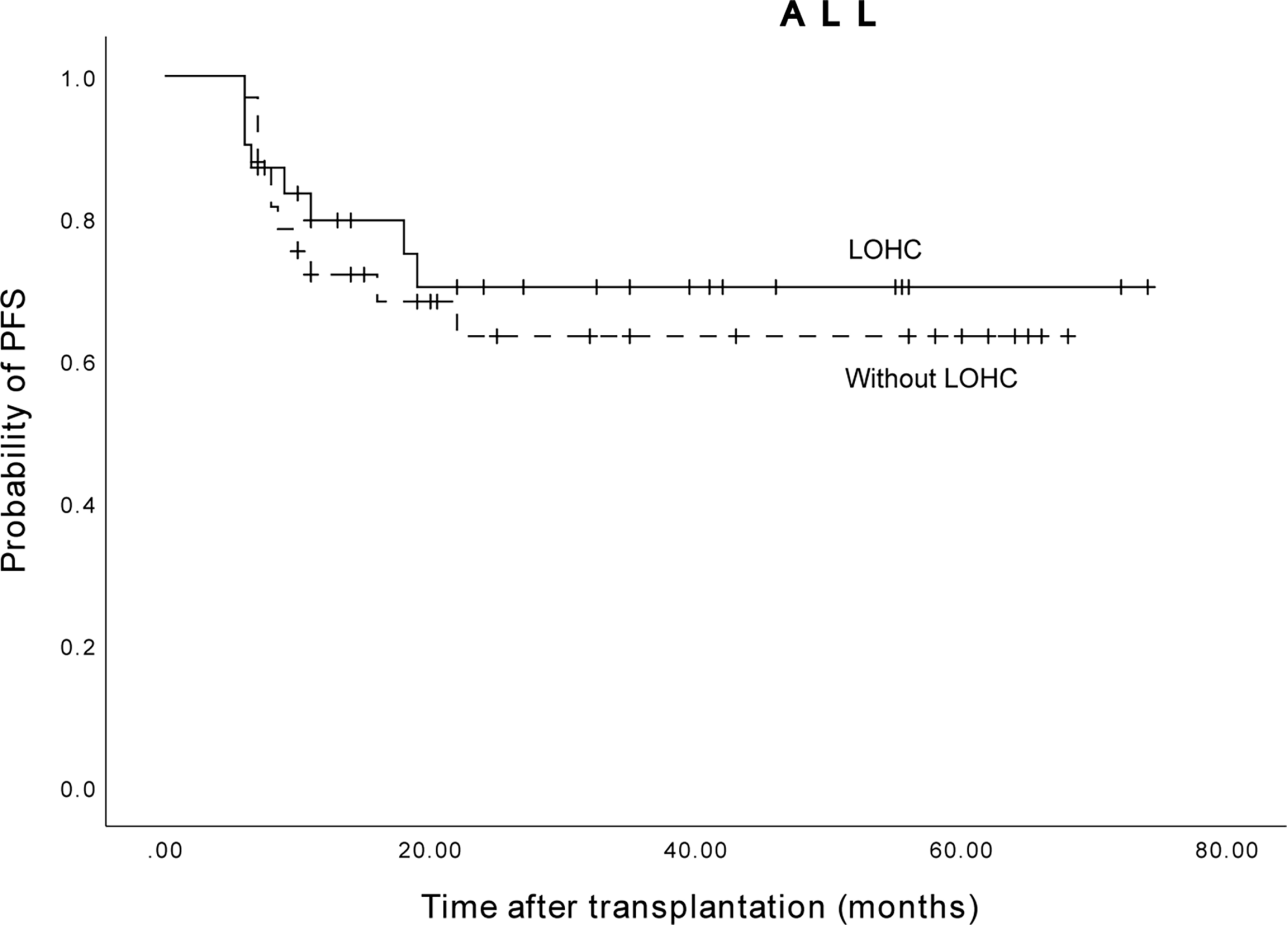
The three years expected PFS of ALL patients with and without LOHC was 63.4% (95%CI, 45.56%~81.24%) and 70.3% (95%CI, 52.46%~88.14%), respectively.

Among the 64 ALL patients, the 3-year expected PFS of 31 patients without LOHC was 70.3% (95% CI, 52.46%~88.14%), and of 33 patients with LOHC was 63.4% (95% CI, 45.56) %~81.24%) (**Figure 7**). The difference was not statistically significant (p=0.849). This suggests that LOHC in ALL patients does not significantly affect the PFS of patients after transplantation.

### Relationship between LOHC duration and PFS of AML and ALL patients

**Figure 8:**
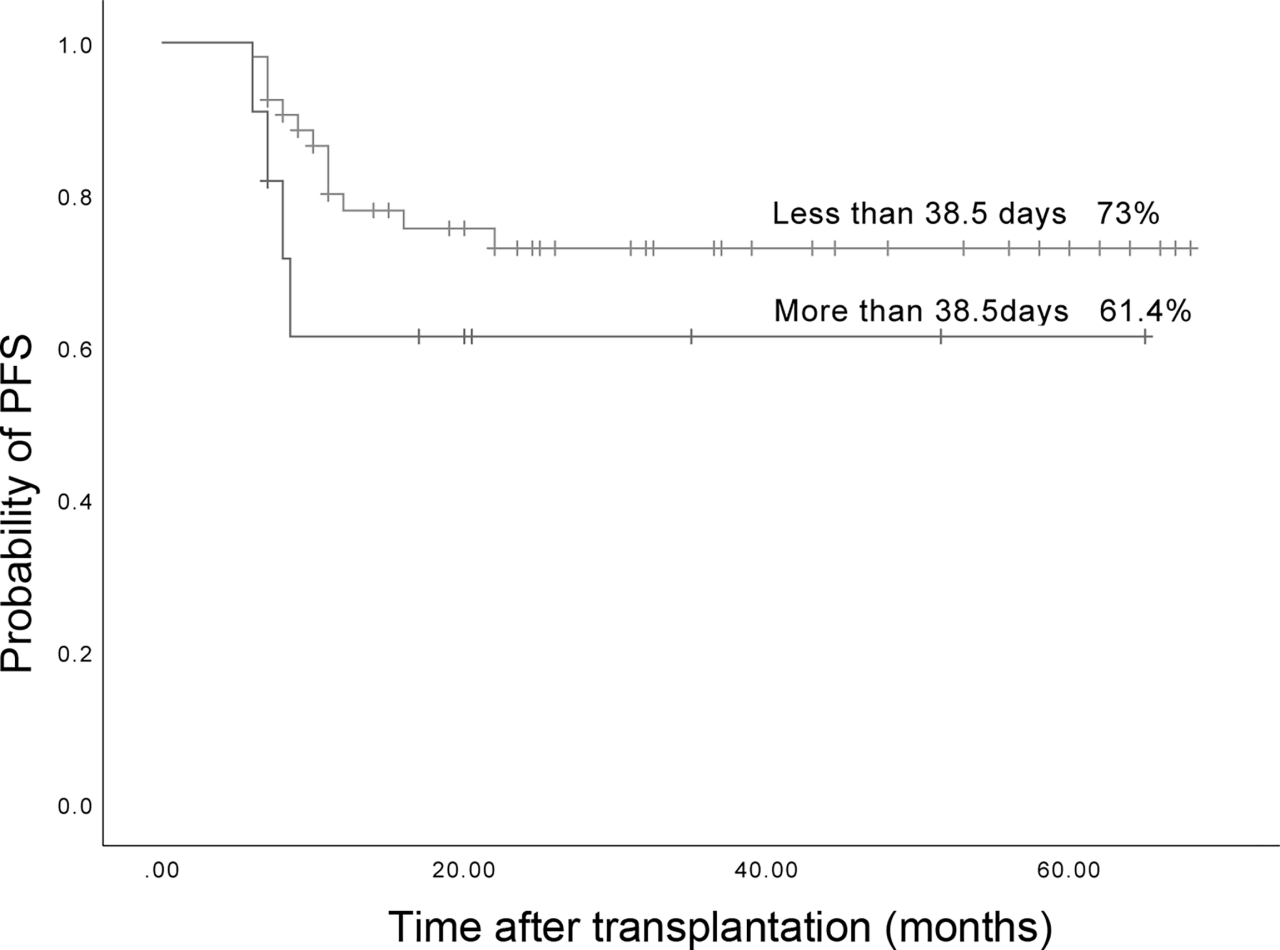
The three years expected PFS in patients with LOHC duration more than 38.5 days and less than 38.5 days was 73% (95%CI, 60.26%~85.74%) and 61.4% (95%CI, 31.42%~91.38%), respectively.

The median duration of LOHC was 38.5 (5-163) days after transplantation. There were 53 patients whose LOHC lasted less than 38.5 days, and 11 patients whose LOHC lasted more than 38.5 days. The 3-year expected PFS of patients with LOHC duration of less than 38.5 days was 73% (95%CI, 60.26%~85.74%), which was not significantly different than that of patients with LOHC duration of more than 38.5 days (61.4% (95%CI, 31.42%~91.38%); p=0.268, **Figure 8**).

Among 31 AML patients with LOHC, 28 patients were with a duration of less than 38.5 days and 3 patients were with a duration of more than 38.5 days. The 3-year expected PFS of patients with LOHC duration of less than 38.5 days was 83.6% (95%CI, 68.61%~98.39%) and that of patients with LOHC duration of more than 38.5 days was 66.7% (95%CI, 43.18%~90.22%). The difference was statistically significant (p=0.048). However, there were fewer cases of AML patients LOHC duration more than 38.5 days. Thus, this result can only indicate that the longer duration of LOHC may affect the PFS of AML patients after transplantation.

In 33 ALL patients with LOHC, 25 patients were with a duration of less than 38.5 days and 8 patients were with a duration of more than 38.5 days. Their 3-year expected PFS was 60.6% (95%CI, 39.82%~81.38%) and 58.3% (95%CI, 43.18%~90.22%), respectively. There was no significant difference (p=0.489).

The above results indicate that the duration of LOHC may not affect the PFS of patients after transplantation.

### Relationship between LOHC severity and PFS of AML and ALL patients

**Figure 9:**
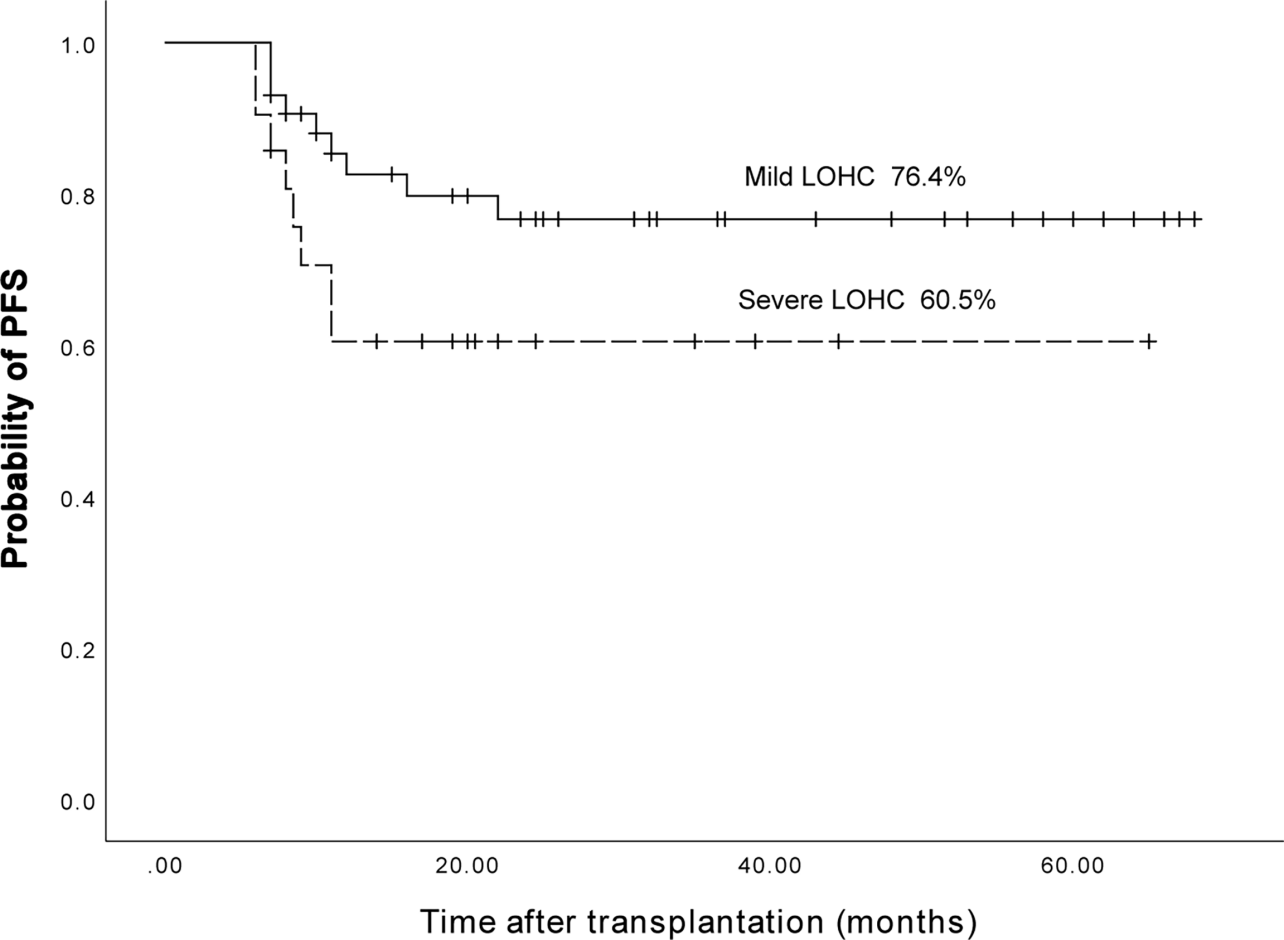
The three years expected PFS in patients with mild LOHC and severe LOHC was 76.4% (95%CI, 63.38%~90.42%) and 60.5% (95%CI, 39.1%~81.9%), respectively.

Among AML and ALL patients, a total of 43 had mild LOHC and 21 had severe LOHC. The 3-year expected PFS of patients with mild LOHC was 76.4% (95%CI, 63.38%~90.42%) and that of patients with severe LOHC 60.5% (95%CI, 39.1%~81.9%) (p=0.08). The difference was not statistically significant (p=0.08, **Figure 9**).

Among 31 AML patients with LOHC, 22 were mild and 9 were severe LOHC. Their 3-year expected PFS was 84.8% (95%CI, 68.92%-100.68%) and 75% (95%CI, 45%~ 105%), respectively. The difference was not statistically significant (p=0.552).

In addition, among 33 ALL patients with LOHC, 21 were mild and 12 were severe. Their 3-year expected PFS was 67.5% (95%CI, 45.55%-89.45%) and 50% (95%CI, 21.78%~78.22%), respectively, without significant difference (p=0.084).

Overall, the severity of LOHC may not affect the PFS of patients after transplantation.

## Discussion

HC is a common complication after allo-HSCT. Viral infection, the strength of the conditioning regimen, calcineurin inhibitors (for the prevention of GVHD), the HLA differences between donors and recipients, and the source of stem cells may all be potential risk factors for the occurrence of HC (16, 17). Mukherjee et al. (17) found that haplo-HSCT was the main risk factor for LOHC occurrence. Lu et al. (18) showed that the occurrence of HC in haplo-HSCT patients was significantly higher than that of HLA-identical transplantation, and they believed that ATG was related to the occurrence of HC. The study by Kerbauy et al. (2) showed that the use of ATG during conditioning significantly increased the incidence of LOHC associated with BK virus infection. Mo et al. (19) reported that the cumulative incidence of LOHC after haplo-HSCT in 149 cases who underwent ATG-based MAC was 29.8%, and the cumulative incidence of severe (grade III-IV) HC was 10.9%. Here, in this single-center retrospective study, the included patients all had acute leukemia, and the types of transplantation were all haploid transplantation. The MAC conditioning regiment was based on ATG and ABuCy. These may reduce the impact of factors such as disease type, pretreatment plan, ATG use and GVHD prevention on the occurrence of LOHC. The cumulative incidence of severe LOHC in our center was relatively low.

Viral infection is generally considered to be the main cause of LOHC. Studies (20, 21) found that adenovirus infection is a major pathogenic factor of LOHC after allo-HSCT. Studies have also shown that the occurrence of LOHC is related to CMV, influenza virus, etc. (22, 23). Arthur et al. (24) described the association between HC and BK virus after bone marrow transplantation for the first time. BK virus is currently widely considered to be one of the main causes of LOHC, and about 4%-50% of LOHC occurrence is related to BK virus (24–28). In this study, we also found that the occurrence of LOHC may be related to BK virus in the urine. Almost all HC patients had positive results for BK virus in the urine, and the virus copy number was relatively high (average 1.92×10^8^ copies/ml). However, there was no significant relationship between BK virus load and the severity and duration of LOHC. In addition, there was no obvious relationship between LOHC and JC virus in the urine. We also found that the BK and JC viruses in the blood were detected in LOHC patients, which suggests that the detection of BK and JC viruses in the blood may not have clinical significance for the diagnosis of LOHC.

Lunde et al. (27) analyzed 1321 (787 males and 534 females) patients who received allo-HSCT, including 219 HC patients, and found that LOHC was more common in males. Lam et al. (29) retrospectively analyzed 661 patients after allo-HSCT and found that male was a risk factor for LOHC. Similarly, Asano et al. (30) indicated that the incidence of LOHC in males was significantly higher than that in females. However, Leung et al. (31) and Seber et al. (32) believed that gender was not related to the occurrence of LOHC. Consistently, in this study, we found that the cumulative incidence of LOHC in female patients was higher than that in males, but the difference was not statistically significant.

Lunde et al. (27) found that LOHC was more common in patients aged 0-20 years old. However, Leung et al. (31) revealed that there was no significant relationship between age and LOHC. Our results showed that younger patients had a higher cumulative incidence of LOHC, especially those under 27 years of age (the median age of LOHC in this study). Therefore, it is necessary to dynamically monitor the BK virus in the urine and clinical symptoms of young patients after allo-HSCT to actively prevent the occurrence of LOHC.

Lee et al. (33) showed that acute GVHD of III ~ IV degree was a risk factor for LOHC. However, the mechanism is still unclear. It is considered that as a target organ, the urothelium may be attacked by GVHD, which causes extensive mucosal damage and severe HC bleeding (33, 34). Salamonowicz-Bodzioch M et al. (34) conducted univariate and multivariate analysis on 133 children undergoing HSCT and found that acute GVHD was a risk factor for HC. They concluded that excessive immune responses and immunosuppressive agents related to acute GVHD may play a key role in HC occurrence. Here, the number of cases in our study was relatively small, and it was difficult to perform a bladder biopsy when the patient had LOHC. Therefore, we cannot clarify the relationship between acute GVHD and LOHC. In our study, the median duration of LOHC was relatively long, mainly because there were 7 severe LOHC patients with a disease course of more than 60 days, including 1 case with LOHC duration of 110 days and 1 case with LOHC duration of 163 days. Among these 7 patients, 5 cases had grade 3 intestinal acute GVHD and 2 cases had grade 2 liver acute GVHD. The acute GVHD was treated with glucocorticoids, MTX or IL-2RAs. The co-occurrence of severe LOHC and acute GVHD suggests that LOHC may be a clinical manifestation of GVHD, or that GVHD may aggravate LOHC. Interestingly, we found that compared with AML patients, the incidence of LOHC was higher in ALL patients. We speculate that this may be related to the higher BK virus infection in ALL patients, or to the earlier reduction of immunosuppressive agents and the higher incidence of GVHD.

Recent studies have shown that although LOHC associated with BK virus infection can increase the risk of TRM, its impact on OS is limited (5, 6, 13, 35). Arai et al. (36) found that there was no statistical difference in OS between patients with and without LOHC. However, compared with mild LOHC (degree I-II) and non-LOHC patients, the OS of severe LOHC (degree III) was significantly lower. Lunde et al. (27) reported that the difference in OS between patients with and without LOHC, nor between patients with different LOHC severity (degree I-II and degree III-IV) was not significantly different. Kerbauy et al. (2) demonstrated that compared with patients without LOHC, the OS of patients with LOHC was significantly affected. Among them, degree III-IV LOHC significantly reduced the survival of patients, while degree I-II LOHC had no significant effect on survival. Overall, severe LOHC may affect the survival of patients. Our results showed that the 3-year expected PFS of patients with and without LOHC was not statistically significant, and the severity of LOHC had no significant effect on PFS. However, in AML patients, the prolonged LOHC duration affected the patients’ PFS. Although no significant effect of LOHC on survival was observed, our results still suggest that timely and effective treatment of severe LOHC is very important to further the potential adverse effect of LOHC on survival of patients.

In this study, there was no death case in 64 patients with LOHC, suggesting that timely and effective treatment may be helpful to improve PFS. Based on our experiences, it is difficult to completely prevent the occurrence of LOHC after haplo-HSCT. Timely treatment such as fluid replacement and diuresis may reduce the progression of mild LOHC to severe LOHC. Our stratified treatment strategy for LOHC patients is as follows: 1) For patients with HC related with BK and other viruses, cidofovir is the first-line treatment with an effective rate of 60%-86% (37–39). In China, however, cidofovir is still not easily available. Therefore, it is necessary to reduce or stop glucocorticoids and other immunosuppressive agents when there is or may be GVHD. Continuous intravenous infusion of human immunoglobulin for 3-4 days to enhance the patient’s non-specific immunity is also beneficial. 2) Studies (40, 41) have shown that aGVHD is a risk factor for the occurrence of LOHC. In our study, some patients had GVHD before the onset of LOHC, especially when there was positive virus infection. When LOHC may be related to GVHD, Methylprednisolone was given to severe LOHC patients, with the treatment course of generally 3-5 days. 3) For patients with refractory and prolonged LOHC, ADSCs infusion was performed. Aronsson-Kurttila et al. (42) treated 11 patients with HC after HSCT with MSC and found that the average disappearance time of hematuria was 22 days. In the study of Wang et al. (43), 7 out of 33 HC patients were treated with MSC, and good results were also achieved. In this study, 5 cases of LOHC patients received ADSCs treatment, all of whom were refractory and protracted. The minimum infusion dose of ADSCs was 0.9×10^6^/kg, and the maximum was 1.4×10^6^/kg, with a minimum of 3 infusions and a maximum of 5 infusions. All 5 patients were cured.

The results of this study showed that after haplo-PBSCT, patients still had a higher incidence of LOHC. Although LOHC had no obvious effect on PFS after transplantation, it is still a potentially life-threatening complication after transplantation. Therefore, timely and effective treatment of LOHC are the basis for promoting the long-term survival of patients. In the future, it is necessary to further explore the relationship between LOHC and PFS, as well as the etiology, pathogenesis and treatment methods of HC, in order to develop a systematic treatment system to further improve the quality of life of patients.

## Data availability statement

The raw data supporting the conclusions of this article will be made available by the authors, without undue reservation.

## Ethics statement

The studies involving human participants were reviewed and approved by the ethics committee of Xinjiang Medical University. The patients/participants provided their written informed consent to participate in this study.

## Author contributions

HY and MJ conceived and designed the experiments. GC, RY, and GA collected the data. HY analyzed the data. JX interpreted the data. HY, GC, and MJ prepared the manuscript. MM searched the literatures. All authors read and approved the final manuscript.

## Funding

This work was supported by the Xinjiang Uygur Autonomous Region Natural Science Fund (No. 2017D01C297). The funder have no role in the design of the study and collection, analysis, and interpretation of data and in writing the manuscript.

## Conflict of interest

The authors declare that the research was conducted in the absence of any commercial or financial relationships that could be construed as a potential conflict of interest.

## Publisher’s note

All claims expressed in this article are solely those of the authors and do not necessarily represent those of their affiliated organizations, or those of the publisher, the editors and the reviewers. Any product that may be evaluated in this article, or claim that may be made by its manufacturer, is not guaranteed or endorsed by the publisher.

## References

[1] Bedi A Miller CB Hanson JL Goodman S Ambinder RF Charache P. Association of BK virus with failure of prophylaxis against hemorrhagic cystitis following bone marrow transplantation. J Clin Oncol (1995) 13:1103–9. doi: 10.1200/JCO.1995.13.5.1103 7738616

[2] Kerbauy LN Kerbauy MN Bautzer V Chapchap EC De Mattos VRP Da Rocha JDA. Severe hemorrhagic cystitis caused by the BK polyomavirus is associated with decreased survival post-allogeneic hematopoietic stem cell transplantation. Transpl Infect Dis (2019) 21:e13101. doi: 10.1111/tid.13101 31054192

[3] Sencer SF Haake RJ Weisdorf DJ. Hemorrhagic cystitis after bone marrow transplantation. risk factors and complications. Transplantation (1993) 56:875–9. doi: 10.1097/00007890-199310000-00020 8212210

[4] Jaiswal SR Singhal P Thatai A Bhagwati G Aiyer HM Chakrabarti A. Impact of extended infusional mesna prophylaxis on the incidence of BK viruria and hemorrhagic cystitis following post-transplantation cyclophosphamide and CTLA4Ig-based haploidentical transplantation. Ann Hematol (2020) 99:839–45. doi: 10.1007/s00277-020-03930-w 32025839

[5] Gilis L Morisset S Billaud G Ducastelle-Lepretre S Labussiere-Wallet H Nicolini FE. High burden of BK virus-associated hemorrhagic cystitis in patients undergoing allogeneic hematopoietic stem cell transplantation. Bone Marrow Transplant (2014) 49:664–70. doi: 10.1038/bmt.2013.235 24488049

[6] Uhm J Hamad N Michelis FV Shanavas M Kuruvilla J Gupta V. The risk of polyomavirus BK-associated hemorrhagic cystitis after allogeneic hematopoietic SCT is associated with myeloablative conditioning, CMV viremia and severe acute GVHD. Bone Marrow Transplant (2014) 49:1528–34. doi: 10.1038/bmt.2014.181 25111517

[7] Cesaro S Dalianis T Hanssen Rinaldo C Koskenvuo M Pegoraro A Einsele H. ECIL guidelines for the prevention, diagnosis and treatment of BK polyomavirus-associated haemorrhagic cystitis in haematopoietic stem cell transplant recipients. J Antimicrob Chemother (2018) 73:12–21. doi: 10.1093/jac/dkx324 29190347

[8] Dropulic LK Jones RJ. Polyomavirus BK infection in blood and marrow transplant recipients. Bone Marrow Transplant (2008) 41:11–8. doi: 10.1038/sj.bmt.1705886 PMC3066131 17952131

[9] Delacruz J Pursell K. BK virus and its role in hematopoietic stem cell transplantation: Evolution of a pathogen. Curr Infect Dis Rep (2014) 16:417. doi: 10.1007/s11908-014-0417-x 24942378

[10] Gander R Asensio M Guillen G Royo GF Bolanos A Perez M. Hemorrhagic cystitis after hematopoietic stem cell transplantation: A challenge for the pediatric urologist. J Pediatr Urol (2018) 14:366–73. doi: 10.1016/j.jpurol.2018.03.018 29776868

[11] Copelan OR Sanikommu SR Trivedi JS Butler C Ai J Ragon BK. Higher incidence of hemorrhagic cystitis following haploidentical related donor transplantation compared with matched related donor transplantation. Biol Blood Marrow Transplant (2019) 25:785–90. doi: 10.1016/j.bbmt.2018.12.142 30579967

[12] Hayden RT Gu Z Liu W Lovins R Kasow K Woodard P. Risk factors for hemorrhagic cystitis in pediatric allogeneic hematopoietic stem cell transplant recipients. Transpl Infect Dis (2015) 17:234–41. doi: 10.1111/tid.12364 PMC4648238 25648430

[13] Abudayyeh A Hamdi A Lin H Abdelrahim M Rondon G Andersson BS. Symptomatic BK virus infection is associated with kidney function decline and poor overall survival in allogeneic hematopoietic stem cell recipients. Am J Transplant (2016) 16:1492–502. doi: 10.1111/ajt.13635 PMC5653306 26608093

[14] Yuan H Chen G Qu J Yang R Muhashi M Aizezi G. Effect of late-onset hemorrhagic cystitis on PFS after haplo-PBSCT. Open Med (Wars) (2021) 16:1493–502. doi: 10.1515/med-2021-0368 PMC8494146 34703902

[15] Brugieres L Hartmann O Travagli JP Benhamou E Pico JL Valteau D. Hemorrhagic cystitis following high-dose chemotherapy and bone marrow transplantation in children with malignancies: incidence, clinical course, and outcome. J Clin Oncol (1989) 7:194–9. doi: 10.1200/JCO.1989.7.2.194 2644398

[16] Rimondo A Crocchiolo R El-Cheikh J Bramanti S Granata A Furst S. The calcineurin inhibitor and the intensity of the conditioning regimen may affect the occurrence of polyomavirus-associated hemorrhagic cystitis after haploidentical hematopoietic stem cell transplantation with post-transplant cyclophosphamide. Bone Marrow Transplant (2017) 52:135–7. doi: 10.1038/bmt.2016.193 27427919

[17] Mukherjee A Milton D Ledesma C Olson A Alatrash G Anderlini P. Influence of the intensity of the allogeneic conditioning regimen on the risk of hemorrhagic cystitis (HC) in patients receiving post- transplant cyclophosphamide (PT-cy) as gvhd prophylaxis. Biol Blood Marrow Transplant (2019) 25:S147–8. doi: 10.1016/j.bbmt.2018.12.441

[18] Lu DP Dong L Wu T Huang XJ Zhang MJ Han W. Conditioning including antithymocyte globulin followed by unmanipulated HLA-mismatched/haploidentical blood and marrow transplantation can achieve comparable outcomes with HLA-identical sibling transplantation. Blood (2006) 107:3065–73. doi: 10.1182/blood-2005-05-2146 16380454

[19] Mo XD Zhang XH Xu LP Wang Y Yan CH Chen H. Treatment of late-onset hemorrhagic cystitis after allogeneic hematopoietic stem cell transplantation: the role of corticosteroids. Ann Hematol (2018) 97:1209–17. doi: 10.1007/s00277-018-3290-0 PMC7080199 29532160

[20] Silva Lde P Patah PA Saliba RM Szewczyk NA Gilman L Neumann J. Hemorrhagic cystitis after allogeneic hematopoietic stem cell transplants is the complex result of BK virus infection, preparative regimen intensity and donor type. Haematologica (2010) 95:1183–90. doi: 10.3324/haematol.2009.016758 PMC2895044 20410183

[21] Gargiulo G Orlando L Alberani F Crabu G Di Maio A Duranti L. Haemorrhagic cystitis in haematopoietic stem cell transplantation (HSCT): a prospective observational study of incidence and management in HSCT centres within the GITMO network (Gruppo italiano trapianto midollo osseo). Ecancermedicalscience (2014) 8:420. doi: 10.3332/ecancer.2014.420 24834115 PMC3998658

[22] Prentice HG Blacklock HA Janossy G Gilmore MJ Price-Jones L Tidman N. Depletion of T lymphocytes in donor marrow prevents significant graft-versus-host disease in matched allogeneic leukaemic marrow transplant recipients. Lancet (1984) 1:472–6. doi: 10.1016/S0140-6736(84)92848-4 6142207

[23] Ringdén O Horowitz MM Sondel P Gale RP Biggs JC Champlin RE. Methotrexate, cyclosporine, or both to prevent graft-versus-host disease after HLA-identical sibling bone marrow transplants for early leukemia? Blood (1993) 81:1094–101. doi: 10.1182/blood.V81.4.1094.1094 8427991

[24] Arthur RR Shah KV Baust SJ Santos GW Saral R. Association of BK viruria with hemorrhagic cystitis in recipients of bone marrow transplants. N Engl J Med (1986) 315:230–4. doi: 10.1056/NEJM198607243150405 3014334

[25] Bogdanovic G Priftakis P Giraud G Kuzniar M Ferraldeschi R Kokhaei P. Association between a high BK virus load in urine samples of patients with graft-versus-host disease and development of hemorrhagic cystitis after hematopoietic stem cell transplantation. J Clin Microbiol (2004) 42:5394–6. doi: 10.1128/JCM.42.11.5394-5396.2004 PMC525183 15528753

[26] Wong AS Chan KH Cheng VC Yuen KY Kwong YL Leung AY. Relationship of pretransplantation polyoma BK virus serologic findings and BK viral reactivation after hematopoietic stem cell transplantation. Clin Infect Dis (2007) 44:830–7. doi: 10.1086/511863 17304456

[27] Lunde LE Dasaraju S Cao Q Cohn CS Reding M Bejanyan N. Hemorrhagic cystitis after allogeneic hematopoietic cell transplantation: risk factors, graft source and survival. Bone Marrow Transplant (2015) 50:1432–7. doi: 10.1038/bmt.2015.162 PMC5343753 26168069

[28] Blackard JT Davies SM Laskin BL. BK polyomavirus diversity-why viral variation matters. Rev Med Virol (2020) 30:e2102. doi: 10.1002/rmv.2102 32128960 PMC7363569

[29] Lam W Storek J Li H Geddes M Daly A. Incidence and risk factor of hemorrhagic cystitis after allogeneic transplantation with fludarabine, busulfan, and anti-thymocyte globulin myeloablative conditioning. Transpl Infect Dis (2017) 19 :e12677. doi: 10.1111/tid.12677 28199755

[30] Asano Y Kanda Y Ogawa N Sakata-Yanagimoto M Nakagawa M Kawazu M. Male Predominance among Japanese adult patients with late-onset hemorrhagic cystitis after hematopoietic stem cell transplantation. Bone Marrow Transplant (2003) 32:1175–9. doi: 10.1038/sj.bmt.1704274 14647272

[31] Leung AY Mak R Lie AK Yuen KY Cheng VC Liang R. Clinicopathological features and risk factors of clinically overt haemorrhagic cystitis complicating bone marrow transplantation. Bone Marrow Transplant (2002) 29:509–13. doi: 10.1038/sj.bmt.1703415 11960271

[32] Seber A Shu XO Defor T Sencer S Ramsay N. Risk factors for severe hemorrhagic cystitis following BMT. Bone Marrow Transplant (1999) 23:35–40. doi: 10.1038/sj.bmt.1701523 10037048

[33] Lee GW Lee JH Choi SJ Kim S Seol M Kim WK. Hemorrhagic cystitis following allogeneic hematopoietic cell transplantation. J Korean Med Sci (2003) 18:191–5. doi: 10.3346/jkms.2003.18.2.191 PMC3055015 12692415

[34] Salamonowicz-Bodzioch M Fraczkiewicz J Czyzewski K Zajac-Spychala O Gorczynska E Panasiuk A. Prospective analysis of BKV hemorrhagic cystitis in children and adolescents undergoing hematopoietic cell transplantation. Ann Hematol (2021) 100:1283–93. doi: 10.1007/s00277-021-04454-7 PMC8043890 33661334

[35] Ruggeri A Roth-Guepin G Battipaglia G Mamez AC Malard F Gomez A. Incidence and risk factors for hemorrhagic cystitis in unmanipulated haploidentical transplant recipients. Transpl Infect Dis (2015) 17:822–30. doi: 10.1111/tid.12455 26354178

[36] Arai Y Maeda T Sugiura H Matsui H Jo T Ueda T. Risk factors for and prognosis of hemorrhagic cystitis after allogeneic stem cell transplantation: retrospective analysis in a single institution. Hematology (2012) 17:207–14. doi: 10.1179/1607845412Y.0000000010 22944099

[37] Philippe M Ranchon F Gilis L Schwiertz V Vantard N Ader F. Cidofovir in the treatment of BK virus-associated hemorrhagic cystitis after allogeneic hematopoietic stem cell transplantation. Biol Blood Marrow Transplant (2016) 22:723–30. doi: 10.1016/j.bbmt.2015.12.009 26718666

[38] Pérez-Huertas P Cueto-Sola M Escobar-Cava P Fernández-Navarro JM Borrell-García C Albert-Marí A. BK virus-associated hemorrhagic cystitis after allogeneic hematopoietic stem cell transplantation in the pediatric population. J Pediatr Oncol Nurs (2017) 34:13–9. doi: 10.1177/1043454216631952 26902502

[39] Aldiwani M Tharakan T Al-Hassani A Gibbons N Pavlu J Hrouda D. BK virus associated haemorrhagic cystitis. a systematic review of current prevention and treatment strategies. Int J Surg (2019) 63:34–42. doi: 10.1016/j.ijsu.2019.01.019 30711618

[40] Peterson L Ostermann H Fiegl M Tischer J Jaeger G Rieger CT. Reactivation of polyomavirus in the genitourinary tract is significantly associated with severe GvHD and oral mucositis following allogeneic stem cell transplantation. Infection (2016) 44:483–90. doi: 10.1007/s15010-016-0872-4 26792012

[41] Coomes EA Wolfe Jacques A Michelis FV Kim DDH Thyagu S Viswabandya A. Efficacy of cidofovir in treatment of BK virus-induced hemorrhagic cystitis in allogeneic hematopoietic cell transplant recipients. Biol Blood Marrow Transplant (2018) 24:1901–5. doi: 10.1016/j.bbmt.2018.04.009 29679772

[42] Aronsson-Kurttila W Baygan A Moretti G Remberger M Khoein B Moll G. Placenta-derived decidua stromal cells for hemorrhagic cystitis after stem cell transplantation. Acta Haematol (2018) 139:106–14. doi: 10.1159/000485735 29408819

[43] Wang Y Chen F Gu B Chen G Chang H Wu D. Mesenchymal stromal cells as an adjuvant treatment for severe late-onset hemorrhagic cystitis after allogeneic hematopoietic stem cell transplantation. Acta Haematol (2015) 133:72–7. doi: 10.1159/000362530 25139500

